# Anxiety promotes memory for mood-congruent faces but does not alter loss aversion

**DOI:** 10.1038/srep24746

**Published:** 2016-04-21

**Authors:** Caroline J. Charpentier, Chandni Hindocha, Jonathan P. Roiser, Oliver J. Robinson

**Affiliations:** 1 Institute of Cognitive Neuroscience, University College London, London WC1N 3AR, UK; 2 Affective Brain Lab, Department of Experimental Psychology, University College London, London WC1H 0AP, UK; 3 Clinical Psychopharmacology Unit, Department of Clinical, Educational and Health Psychology, University College London, WC1E 7HB.

## Abstract

Pathological anxiety is associated with disrupted cognitive processing, including working memory and decision-making. In healthy individuals, experimentally-induced state anxiety or high trait anxiety often results in the deployment of adaptive harm-avoidant behaviours. However, how these processes affect cognition is largely unknown. To investigate this question, we implemented a translational within-subjects anxiety induction, threat of shock, in healthy participants reporting a wide range of trait anxiety scores. Participants completed a gambling task, embedded within an emotional working memory task, with some blocks under unpredictable threat and others safe from shock. Relative to the safe condition, threat of shock improved recall of threat-congruent (fearful) face location, especially in highly trait anxious participants. This suggests that threat boosts working memory for mood-congruent stimuli in vulnerable individuals, mirroring memory biases in clinical anxiety. By contrast, Bayesian analysis indicated that gambling decisions were better explained by models that *did not* include threat or treat anxiety, suggesting that: (i) higher-level executive functions are robust to these anxiety manipulations; and (ii) decreased risk-taking may be specific to pathological anxiety. These findings provide insight into the complex interactions between trait anxiety, acute state anxiety and cognition, and may help understand the cognitive mechanisms underlying adaptive anxiety.

Anxiety disorders constitute a major global health burden^1^, and are characterized by negative emotional processing biases, as well as disrupted working memory and decision-making^2^,^3^. On the other hand, anxiety can also be an adaptive response to stress, stimulating individuals to engage in harm-avoidant behaviours. Influential theories of pathological anxiety propose that clinical anxiety emerges through dysregulation of adaptive anxiety^4^,^5^. Therefore, in order to understand how this dysregulation emerges in pathological anxiety, it is crucial to first understand the cognitive features associated with adaptive or ‘non-pathological’ anxiety, in other words anxiety levels that can vary within and between individuals but do not result in the development of clinical symptoms associated with anxiety disorders.

Several methods exists to induce anxiety in healthy individuals, including threat of shock (ToS), the Trier social stressor test (TSST), and the cold pressor test (CPT). During the ToS paradigm, subjects typically perform a cognitive task while either at risk of or safe from rare, but unpleasant, electric shocks. Compared to the other methodologies, ToS has the advantage of allowing for within-subjects, within-sessions, designs (for a review on its effects on cognition, see Robinson *et al*.^2^), and ensures the task is performed while being anxious, rather than after being relieved from the stressor. In addition, ToS paradigms have good translational analogues^6^, are well-validated^7^, and are thus considered a reliable model for examining adaptive anxiety in healthy individuals.

Because the engagement of adaptive anxiety processes may vary with individuals’ vulnerability to developing pathological anxiety^8^,^9^,^10^, we were also interested in examining how the effects of state anxiety induced by threat of shock interact with dispositional or trait anxiety, as reflected in self-report questionnaire scores such as the State-Trait Anxiety Inventory^11^ (STAI). High levels of self-reported trait anxiety are indeed considered a strong vulnerability factor in the development of pathological anxiety^4^,^12^.

The extent to which induced state anxiety (elicited by the laboratory procedures discussed above) and trait anxiety interact to alter cognition has rarely been studied^10^. In particular, does induced anxiety have a disproportionate impact on cognition in those individuals with high trait anxiety, and thus greater proneness to pathological anxiety? To answer this question, in the present study we combined a ToS paradigm with a task that concomitantly measures emotional working memory and economic decision-making^9^, testing healthy individuals with a wide range of trait anxiety scores.

Using this task, the aim of this study was to therefore test the impact of 1) induced (state) and 2) dispositional (trait) anxiety on a) working memory and b) decision-making.

Anxiety has been associated with working memory impairment, including anxiety induced by ToS^13^,^14^, individuals with high dispositional anxiety^13^,^15^, and clinical anxiety^16^,^17^. However, this has yet to be investigated using emotional stimuli, which are highly salient, and for which working memory is therefore likely to be enhanced regardless of anxiety^18^. People with high trait anxiety exhibit an enhanced response to negative stimuli, such as fearful faces, in attentional tasks^2^,^19^,^20^,^21^, consistent with a mood-congruent bias in the allocation of attentional resources.

Anxiety is also associated with important difficulties in making decisions. When faced with risky decisions, individuals with an anxiety disorder (see Table 1A for a summary of studies) or healthy individuals with high dispositional anxiety^22^,^23^,^24^,^25^ exhibit increased risk avoidant behaviour. However, evidence for an effect of acute state anxiety on risky decision-making is very mixed; the presence and direction of effects appear to vary substantially depending on the induction technique, the decision-making task, and individual differences in trait anxiety, age and gender (see Table 1B for a summary of studies).

Clark *et al*.^26^ found increased risk averse choices on a gambling task under threat, but only when the risky option contained a moderate loss or when both choice options involved only losses. When the risky option involved a high loss or when choosing between gain-only options, ToS had no effect on risk taking^26^. However, in this study, threat was not sustained, occurring only for short durations (5–5.5 s), making it more comparable to a fear-cueing paradigm than an anxiety induction. More recently, ToS was shown to interact with individual levels of trait anxiety to alter decision making on the Iowa Gambling Task, such that low anxious individuals took fewer risks under threat while high anxious individuals took more risks^10^. Most of the other studies listed in Table 1B did not examine individual differences in trait anxiety, so this is a possible explanation for the mixed evidence of induced stress on risky decisions. In addition, several studies suggest differential effects of stress in the gain and in the loss domain, suggesting that stress could act on risky decisions by altering loss aversion – the relative weighting of losses over gains. Surprisingly, whether loss aversion is affected by induced anxiety has never been tested.

These findings led to a number of predictions. Firstly we predicted that both working memory and decision-making would be influenced by threat of shock. Specifically we hypothesized that a mood-congruent bias would be present for emotional working memory and enhanced by ToS^19^,^21^. With respect to decision-making, clinical findings^23^,^24^,^27^ and the results of Clark *et al*.^26^ led us to predict that loss aversion would increase under ToS. In addition based on Robinson *et al*.^10^ and Charpentier *et al*.^9^, we predicted that these state anxiety induced changes would vary as a function of trait anxiety levels, with the most profound effects in those with high trait anxiety.

## Results

**Figure 1:**
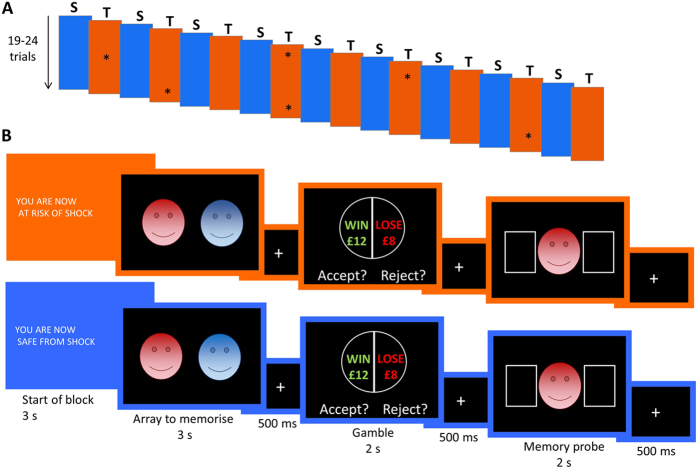
Experimental design. (**A**) Task structure: 18 blocks were segregated into 9 safe (blue) and 9 threat (orange) alternating blocks. Each block lasted between 19–24 trials, the sum of which was 196 trials for safe and for threat. This was to ensure participants could not count the block length. The initial safe or threat block was counterbalanced across participants. Asterisks represent where in each threat block a shock was delivered, if at all. Shocks could be delivered during the first or second fixation cross of the trial (this was also randomized). Six shocks were delivered across the 9 threat blocks, such that the threat that a shock could happen at any time was still present, and participants could not know for sure that after receiving a shock no other shock would follow in that block. In total there were 49 trials per emotion. (**B**) Trial structure: at the beginning of each block participants were informed whether they were in a threat or safe block; throughout the block the corresponding color (threat: orange; safe: blue) was maintained in the frame of the screen. On each trial they were presented with a pair of faces or objects to memorize for 3 seconds. Note that the figure is schematic, in the task we used pictures of real faces from the NimStim face stimulus set (http://www.macbrain.org/resources.htm), and pictures of light bulbs as objects. After a short fixation cross, they then had to decide whether to accept or reject a mixed gamble with 50% chance to win the amount in green and 50% chance to lose the amount in red. Finally, they had to indicate the position in which the probe face/object had been on the first screen.

Fifty-five participants (see Table 2 and Methods for more details) completed a task designed to measure emotional influences on both working memory and economic decision-making (in particular loss aversion). A similar version of this task was described previously^9^ and was adapted in the present experiment to include a ToS manipulation. The task consisted of 18 blocks, with participants completing nine threat blocks alternating with nine safe blocks (Fig. 1A). Whether the first block was safe or threat was counterbalanced across subjects. The trial structure is presented in Fig. 1B.

On each trial, participants were initially instructed to memorize the locations of two prime stimuli, both belonging to one of the following four conditions: happy faces, fearful faces, neutral faces, or objects (light bulbs). Participants then had to decide whether to accept or reject a risky gamble consisting of 50% chance to win the amount in green and 50% chance to lose the amount in red. Finally, one of the initial prime stimuli from the pair appeared (memory probe), and participants were asked to indicate whether it had been located on the left or the right of the first screen.

To investigate the effect of ToS, emotional condition, and trait anxiety on both working memory and gambling behaviour, the following variables were computed and analysed, using both a frequentist and a Bayesian approach (see Methods for details): working memory accuracy and reaction times, proportion of gambles accepted, gambling decision reaction times, choice parameters extracted from Prospect Theory model, namely loss aversion and choice consistency^38^,^39^,^40^. Summary data from these variables are presented in Table 3, and detailed data are available for download (https://dx.doi.org/10.6084/m9.figshare.3160498.v1).

### Manipulation checks

#### State anxiety

To check that the ToS manipulation produced the expected effects on state anxiety, participants completed the Short State Anxiety Inventory^41^ (SSAI) before starting the practice tasks and after completing the main task, retrospectively for the safe condition and for the threat condition (see Table 2 for mean SSAI scores). They also answered the following 3 questions at the end of the study: “How anxious were you in the safe/threat condition?”, “How afraid were you in the safe/threat condition?”, and “How stressed were you in the safe/threat condition?” on a scale from 1 (not at all) to 10 (very much so). Main effects of threat on the different ratings were analysed in repeated-measures ANOVAs, with mean centered trait anxiety added as a covariate. As expected there was a significant main effect of threat on SSAI (measured pre-task, post-task for safe, and post-task for threat: *F*(2,106) = 69.98, *p* < 0.001, *η*_*p*_^*2*^ = 0.569). Participants retrospectively rated themselves as feeling significantly more anxious under threat in comparison to safe (t(53) = 9.38, *p* < 0.001). In comparison to baseline (before the start of the experiment) participants rated themselves as significantly more anxious during the threat condition (threat vs baseline: t(53) = 10.60, *p* < 0.001), while state anxiety levels did not differ significantly between baseline and the safe condition (safe vs baseline: t(53) = 1.48, *p* = 0.144). Similar results were found for single item retrospective ratings of ‘anxious’ (safe: M = 2.69, SD = 2.07; threat: M = 5.80, SD = 2.06; *F*(1,53) = 116.3, *p* < 0.001, *η*_*p*_^*2*^ = 0.687), ‘stressed’ (safe: M = 2.15, SD = 1.43; threat M = 5.65, SD = 2.39; *F*(1,53) = 124.5, *p* < 0.001, *η*_*p*_^*2*^ = 0.701) and ‘afraid’ (safe: M = 1.69, SD = 1.30; threat M = 5.13, SD = 2.11; *F*(1,53) = 159.6, *p* < 0.001, *η*_*p*_^*2*^ = 0.751). Note that except for baseline rating on the SSAI, which was obtained before the experiment, all ratings relating to the threat and safe conditions were collected retrospectively after the end of the task.

#### Trait anxiety

Interestingly, the ToS manipulation was more effective in high anxious individuals. The increase in SSAI during threat blocks relative to baseline and safe blocks interacted with trait anxiety (threat by trait anxiety interaction: F(2,106) = 3.279, *p* = 0.042, *η*_*p*_^*2*^ = 0.058), such that high trait anxious individuals showed a greater threat-induced increase in state anxiety than low trait anxious individuals. This means that any effect of trait anxiety on memory or decision-making could be driven by individual differences in state anxiety induced by the stress manipulation. To account for this potential confound, the change in reported state anxiety between the threat and safe blocks was mean-corrected and added as an additional covariate in the ANOVAs in which an interaction with trait anxiety was identified.

### Working memory performance: mood-congruent effects of threat

Working memory accuracy scores for each condition are reported in Table 3, and were analyzed after arcsine transformation in a 2 (threat: threat, safe) by 4 (emotion: happy, fearful, neutral, object) repeated-measures ANOVA, with trait anxiety added as a covariate.

#### State anxiety

There was no main effect of threat on working memory performance (*F*(1,53) = 1.54, *p* = 0.22, *η*_*p*_^*2*^ = 0.028). There was a main effect of emotion (*F*(3,159) = 4.846, *p*=0.003, *η*_*p*_^*2*^ = 0.084), as well as a significant emotion-by-threat interaction (*F*(3,159) = 3.311, *p* = 0.022, *η*_*p*_^*2*^ = 0.059), driven by worse memory performance for the object condition, especially under threat, suggesting that the emotional content of faces *per se* does not alter the recall of their location. A Bayesian repeated-measures ANOVA confirmed these results, with substantial evidence against a main effect of threat (Bayes Factor representing evidence for the model of interest over the null model: BF_10_ = 0.176) and for a main effect of emotion (BF_10_ = 9.74). However, adding the emotion-by-threat interaction to a model already containing a main effect of emotion, did not improve the model (BF_10_ ratio between the two models = 0.945).

#### Trait anxiety

**Figure 2:**
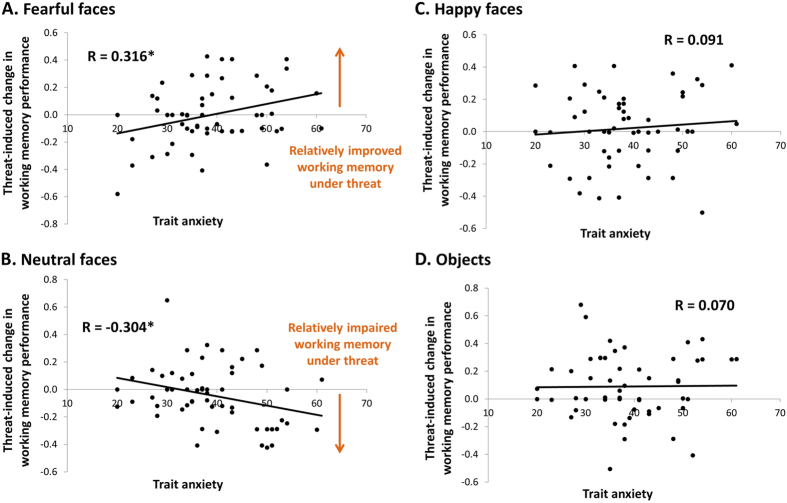
Threat-induced changes in working memory. Differences in working memory performance (arcsine-transformed proportion correct) between threat and safe blocks were calculated for each emotion condition and correlated with trait anxiety. Trait anxiety was associated with (**A**) a threat-induced improvement in working memory for fearful faces, such that highly anxious participants were more accurate in remembering the location of a fearful face under threat; and (**B**) with a threat-induced impairment in working memory for neutral faces, such that highly anxious participants were less accurate in remembering the location of a neutral face under threat. There was no association between trait anxiety and threat-induced changes in working memory for happy faces (**C**) and objects (**D**) condition. *p < 0.05, two-tailed Pearson correlation.

There was a significant emotion-by-threat-by-trait anxiety interaction on working memory accuracy (*F*(3,159) = 3.34, *p* = 0.021, *η*_*p*_^*2*^ = 0.059), as well as a significant two-way interaction between emotion and trait anxiety (*F*(3,159) = 3.715, *p* = 0.013, *η*_*p*_^*2*^ = 0.065), but no main effect of trait anxiety (*F*(1,53) = 0.179, *p* = 0.67, *η*_*p*_^*2*^ = 0.003). Importantly, the interactions with trait anxiety remained significant after controlling for the change in self-reported state anxiety after threat relative to safe blocks (emotion-by-trait anxiety interaction: *F*(3,156) = 2.747, *p* = 0.045, *η*_*p*_^*2*^ = 0.050; emotion-by-threat by-trait anxiety interaction: *F*(3,156) = 3.885, *p* = 0.010, *η*_*p*_^*2*^ = 0.070). Post-hoc analysis of the three-way interaction revealed a significant positive correlation between trait anxiety and threat-induced improvement in working memory for fearful faces (*r*(55) = 0.316, *p* = 0.019, Fig. 2A). Interestingly, there was also a significant negative correlation between trait anxiety and threat-induced change in working memory for neutral faces (*r*(55) = −0.304, *p* = 0.024, Fig. 2B). These correlations differed significantly (Steiger’s Z = 3.34, p < 0.001) and there was no correlation between trait anxiety and threat-induced change in memory performance for happy faces (*r*(55) = 0.091, *p* = 0.51, Fig. 2C) and for objects (*r*(55) = 0.010, *p* = 0.61, Fig. 2D). In other words these results suggest that threat improves working memory accuracy for fearful faces in highly anxious individuals; however, highly anxious individuals had relatively impaired working memory for neutral faces when under threat. A Bayesian test was performed to corroborate this effect. In particular the correlation between trait anxiety and the difference in working memory accuracy for fearful relative to neutral faces between threat and safe conditions (*r*(55) = 0.448) revealed a BF_10_ of 51.6, indicative of very strong evidence in favour of this interaction, relative to the null model.

### Propensity to gamble is unaffected by anxiety

Gambling scores for each condition are reported in Table 3. On average, participants accepted the gamble on 47.14% of trials (SD = 11.76), suggesting that the tailoring procedure to create the gamble matrix according to each participant’s indifference point had operated effectively. With this maximized sensitivity, we then investigated whether propensity to gamble was modulated by the experimental conditions by performing a similar repeated-measures ANOVA to the one described above.

#### State anxiety

There was no main effect of threat (*F*(1,53) = 0.026, *p* = 0.87) and no interactions reached significance (all *Fs* < 1.1, all *Ps* > 0.35). We did find a significant main effect of emotion (*F*(3,159) = 4.574, *p* = 0.004, *η*_*p*_^*2*^ = 0.079); however, this effect was entirely driven by a greater propensity to gamble in the object condition relative to the fearful (t(54) = 3.22, *p* = 0.002) and neutral (t(54) = 2.90, *p* = 0.005) conditions. These results were confirmed by the Bayes Factor analysis revealing that the null model performed substantially better (9.2 times) than a model including only a main effect of threat (BF_10_ = 0.108), suggesting that gambling is not affected by ToS. Consistent with the main effect of emotion evidenced above, the Bayesian analysis also showed that a model including only a main effect of emotion performed strongly better (BF_10_ = 17.42) than the null model. However, this effect was again driven by the object condition, suggesting that the emotional content of faces *per se* do not influence gambling decisions.

#### Trait anxiety

There was no main effect of trait anxiety (*F*(1,53) = 0.193, *p* = 0.66) and no interaction with trait anxiety that reached significance (all *Fs* < 1.1, all *Ps* > 0.35) when analyzing propensity to gamble data. These results were confirmed by the Bayes Factor analysis revealing that the null model performed strongly better (23.5 times) than a model that included main effects of threat, emotion, trait anxiety, and a threat-by-emotion interaction (BF_10_ = 0.043).

### Choice parameters: loss aversion and choice consistency are also unaffected by anxiety

Modelling loss aversion and choice consistency from participants’ choice data (see Methods for details of the modelling procedure) provided a more fine-grained insight into individual differences in gambling behavior. The Prospect Theory-derived model of choice was estimated successfully in all 55 participants (mean R^2^ = 0.656, range = 0.27–0.91). Estimated for each participant across all trials, mean loss aversion was 1.92 (SD = 1.31, range = 0.60–7.14, see Table 3 for data in each condition), consistent with previous literature suggesting that people weight losses about twice as much as gains when making a decision^40^,^42^,^43^,^44^. Mean choice consistency was 2.35 (SD = 1.65, range = 0.34–6.0, see Table 3 for data in each condition).

#### State anxiety

No significant main effects of threat, emotion or threat-by-emotion interaction were identified for loss aversion (all *Fs* < 1.4, all *Ps* > 0.25) or choice consistency (all *Fs* < 1.8, all *Ps* > 0.16) in the repeated-measures ANOVA. These null effects were confirmed by Bayes Factor analysis. For loss aversion, the null model performed substantially (8.9 times) better than the model including only a main effect of threat (BF_10_ = 0.112) and strongly (14.5 times) better than a model including only a main effect of emotion (BF_10_ = 0.069). For choice consistency, the null model also performed substantially (9.5 times) better than the model including only a main effect of threat (BF_10_ = 0.105) and strongly (10.6 times) better than a model including only a main effect of emotion (BF_10_ = 0.094). These analyses suggest that loss aversion and choice consistency are not affected by threat or incidental emotional primes.

#### Trait anxiety

No significant main effects of or interactions with trait anxiety were identified for loss aversion (all *Fs* < 1.4, all *Ps* > 0.25) or choice consistency (all *Fs* < 1.8, all *Ps* > 0.16) in the threat-by-emotion-by-trait anxiety ANOVA. Again these null effects were confirmed by Bayes Factor analysis. For loss aversion, the null model performed decisively (1,896 times) better than the full model (threat + emotion + threat-by-emotion + trait anxiety: BF_10_ = 0.00053). For choice consistency, the null model performed decisively (6,747 times) better than the full model (threat + emotion + threat-by-emotion + trait anxiety: BF_10_ = 0.00015). These analyses suggest that loss aversion and choice consistency are not additionally affected by trait anxiety.

### Working memory reaction times: threat induces a general speeding of responses

A similar ANOVA (with emotion, threat and trait anxiety as factors) was run on reaction times to indicate the location of the memory probe (see Table 3 for reaction times in each condition).

#### State anxiety

**Figure 3:**
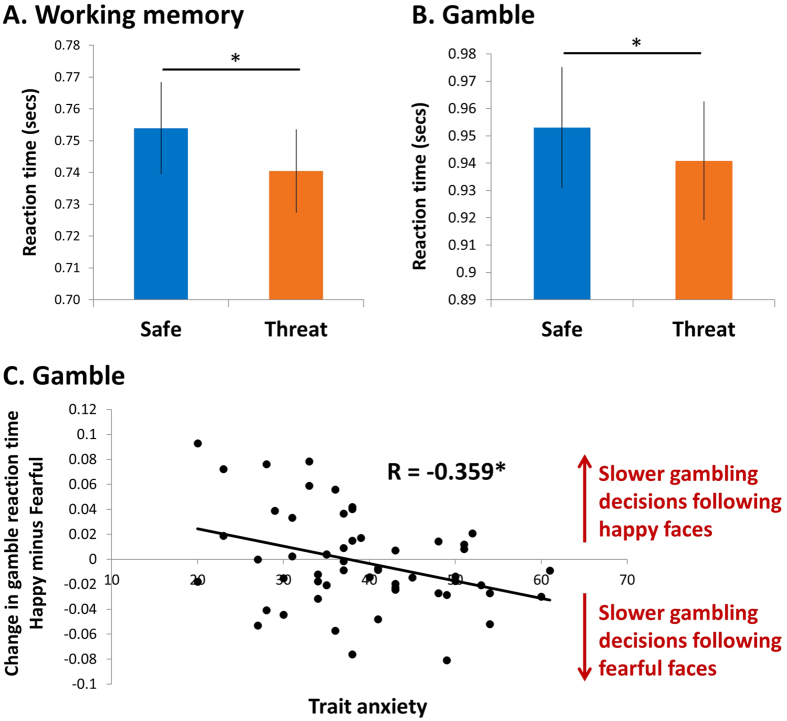
Effects of threat, emotion and trait anxiety on reaction times. Both working memory (**A**) and gamble (**B**) responses are speeded by threat. (**C**) The difference in reaction times to make an economic decision between happy and fearful prime conditions correlates negatively with trait anxiety across individuals. Highly anxious individuals make slower decisions following fearful faces. Error bars represent SEM. *p < 0.05.

We found a main effect of threat (*F*(1,53) = 5.051, *p* = 0.029, *η*_*p*_^*2*^ = 0.087), such that participants were faster overall under threat relative to safe (Fig. 3A). There was also a significant main effect of emotion (*F*(1,53) = 11.26, *p* < 0.001, *η*_*p*_^*2*^ = 0.175), driven by slower responses in the object condition (mean ±SD = 772 ms ±104) relative to all face conditions (happy = 738 ms ±98, t(54) = 4.32; fearful = 741 ms ±109, t(54) = 3.85; neutral = 739 ms ±107, t(54) = 4.62; all *p* < 0.001).

Bayesian analysis confirmed that the best model of working memory reaction times included a main effect of threat, as well as a main effect of emotion (ln(BF_10_) = 12.51) indicative of decisive evidence for this model relative to the null model. This model was also very strongly (39.3 times) better than a model additionally including the threat-by-emotion interaction (ln(BF_10_) = 8.84).

#### Trait anxiety

We did not observe an interaction with or a main effect of trait anxiety on working memory reaction times (all *Fs* < 1.1, all *Ps* > 0.4). Bayesian analysis confirmed that adding trait anxiety to a model already including main effects of threat and emotion did not improve the model (BF_10_ ratio between the two models = 0.559).

### Gambling decision reaction times

A similar ANOVA was used to analyze reaction times for gambling decisions (see Table 3 for reaction times in each condition), with gamble choice (whether gamble is accepted or rejected) as an additional factor.

#### State anxiety

Participants were significantly faster to decide whether to gamble or not under threat (main effect of threat: *F*(1,53) = 7.07, *p* = 0.010, *η*_*p*_^*2*^ = 0.118, Fig. 3B). They were also faster at deciding to accept a gamble than at deciding to reject it (main effect of gamble choice: *F*(1,53) = 4.567, *p* = 0.037, *η*_*p*_^*2*^ = 0.079). No other main or interaction effects reached significance (all *Fs* < 2.1, all *Ps* > 0.1). These results were corroborated using Bayes Factor analysis. A model including both a main effect of threat and a main effect of gamble choice on gambling reaction times performed decisively better than the null model (ln(BF_10_) = 11.73). These results on gambling reaction times suggest that regardless of trait anxiety participants were faster to make economic decisions under threat.

#### Trait anxiety

We found a significant emotion-by-trait anxiety interaction (*F*(3,159) = 3.219, *p* = 0.024, *η*_*p*_^*2*^ = 0.057) on gambling decision reaction time. This interaction was driven by a negative correlation between trait anxiety and the difference in gamble reaction time between happy and fearful conditions (*r* = −0.359, *p* = 0.007, Fig. 3C). No other interaction effects or main effect of trait anxiety reached significance (all *Fs* < 2.1, all *Ps* > 0.1). Bayes Factor analysis showed that a model with a correlation between trait anxiety and reaction time difference between happy and fearful conditions performed substantially (5.71 times) better than the null model. This suggests that high anxious individuals were slower to make economic decisions specifically when primed with fearful faces.

## Discussion

The present study demonstrates that while ToS and emotion interact to alter working memory performance in a trait anxiety-dependent fashion, gambling decisions, and particularly loss aversion, are largely robust to these manipulations and do not vary with trait anxiety in our sample of healthy subjects. We also identified a general speeding of both gambling and working memory responses during ToS.

We found that highly *trait* anxious people under high *state* anxiety (induced by ToS) exhibit improved working memory for the location of fearful faces. This pattern was reversed for neutral faces. This suggests a threat-potentiated improvement in working memory for mood-congruent stimuli: while high trait anxious people memorise fearful faces better when their state anxiety is also high, low anxious people memorise neutral faces better when their state anxiety is high. This interpretation is in line with findings that high state anxiety can enhance processing of affectively-congruent stimuli, in this case fearful faces that convey the most relevant signal to individuals anticipating an aversive shock^19^,^20^,^45^. Interestingly, we additionally show in this study that this enhancement is specific to those individuals who exhibit high dispositional anxiety. This could be explained by improved perceptual sensitivity for fearful cues under ToS in high trait anxious individuals^46^, and may indicate that high *trait* anxiety boosts the selectivity of threat-induced *state* anxiety^47^.

Despite these effects on working memory accuracy for fearful faces, contrary to predictions no effect of threat or interaction with the emotional content of face primes were observed on gambling decisions. We observed this absence of effect in two analyses; using a simple analysis of participants’ propensity to gamble, as well as a Prospect Theory-derived modelling approach to estimate loss aversion and choice consistency. Using Bayesian analysis, our results provide substantial to strong evidence that loss aversion (i) does not vary with trait anxiety (consistent with Ernst *et al*.^28^), and (ii) is robust to state anxiety and emotional manipulation (consistent with Engelmann *et al*.^48^ and Robinson *et al*.^37^). We initially hypothesized that loss aversion would either increase under ToS (as predicted by Clark *et al*.^26^) or vary under ToS as a function of individual levels of trait anxiety (as predicted by Charpentier *et al*.^9^ and Robinson *et al*.^10^), but this was not established. In fact Bayesian analysis revealed the best model of loss aversion to be the null model; with lower evidence for models including incidental emotion, induced state anxiety and trait anxiety, indicating that none of these factors impact loss aversion. A potential limitation that could explain the absence of effects is the fact that our gambling task only included mixed gambles (between a loss and a gain), but not gain-only or loss-only gambles. This prevented us from distinguishing between loss aversion and risk preference in our Prospect Theory model. While loss aversion may be unaffected by state or trait anxiety, it is possible that risk preference in either the gain and/or in the loss domain, could be sensitive to these factors. We also observed that propensity to gamble was reduced following the incidental presentation of face relative to object stimuli, suggesting that exposition to faces, regardless of emotional expression, may result in more conservative decisions and less risk-taking. However, this effect was not present when examining loss aversion parameters, and should therefore be taken with caution.

Explicit decisions were not impacted by trait anxiety, induced state anxiety or incidental emotion; however we find that decision reaction times were affected, suggesting a more implicit effect of threat-induced state anxiety and emotional cues on gambling decisions. First, we found that reaction times to make these gambling decisions, but also reaction times to remember the location of the memory probe, were faster under threat. This threat-induced speeding of subsequent decisions has been observed in recent risky decision-making tasks performed under threat^26^,^37^,^48^, despite the absence of effects of threat on decisions. However, the fact that this speeding in reaction times is also present for the memory task suggests a more general threat-induced and task-independent speeding of cognitive processes.

Second, we also demonstrate that reaction times to gamble are altered by the emotional content of the stimuli preceding the gamble in a manner that depends on trait anxiety. Specifically, high anxious individuals were slower to decide whether to gamble after seeing fearful faces; while low anxious individuals were slower to make decisions after seeing happy faces. This finding may be explained by greater distractibility by affectively-congruent stimuli. In particular, there is clear evidence that high anxious individuals are more distracted by task-irrelevant fearful faces compared to low anxious individuals^49^,^50^,^51^,^52^. According to this explanation, high anxious individuals have slowed responses because they preferentially attend to (and are distracted by) fearful faces, thus making engaging with the gambling task more effortful. Note that this effect was not affected by ToS, suggesting that while state anxiety influences the memory encoding of emotional faces, it does not interact with the impact of emotional faces on task-irrelevant processes.

The fact that we demonstrate clear effects of state and trait anxiety on working memory, but not on higher order decision-making, may be because threat specifically alters lower level, bottom-up processes, such as encoding of and perceptual sensitivity to fearful faces^20^,^46^, while leaving higher order, top-down processes intact^37^. Interestingly, Engelmann *et al*.^48^ showed that despite an absence of threat-effects on loss aversion, the neural encoding of gain and loss value in ventromedial prefrontal cortex, ventral striatum, and insula changed under threat. It is possible that while these lower level valuation systems in the brain were altered by threat-induced state anxiety, the more “deliberative” processes (such as value integration and comparison) were not, resulting in unchanged gambling decisions. This mechanism – state anxiety altering low-level, but not high-level processes – may be adaptive, as it can improve detection and adaptation to changes in the environment while preserving the ability to make more deliberative, controlled, decisions even in anxiogenic environments.

Potential improvements in future studies could include an objective, physiological measure of state anxiety induced by threat of shock (e.g. skin conductance response), to ensure consistency with self-reported state anxiety. Nonetheless, it should be emphasized from extensive prior work that threat of shock has been shown to reliably and concomitantly increase self-report^2^, physiological (e.g. startle response^7^) and neural^6^,^20^ markers of anxiety.

Taken together, our findings provide insights into how state anxiety and trait anxiety interact to alter cognition. They also provide a framework to better understand the cognitive mechanisms by which ‘non-pathological’ anxiety may be adaptive.

## Methods

### Participants

Fifty-six participants took part in this experiment; however, trait anxiety and personality data were not available for one participant and analyses are therefore reported for n = 55 participants (31 female: 24 male; mean age = 24.15, SD = 5.59, see Table 2 for demographics and questionnaire scores). Based on effect sizes from previous related work (d = 0.45 for a meta-analysis of threat-related attentional biases in anxiety^21^, d = 0.40 for the decrease in gambling behavior under ToS^26^), a power calculation using G*power (version 3.1.9.2)^53^ revealed that a sample size of 55 would achieve between 83% and 90% power to detect such effects with an alpha of 0.05. Ethical approval was obtained from the UCL Research Ethics Committee and the study was carried out in accordance with the approved protocol. Participants were recruited via responses to an advertisement through institutional mailing lists and provided written, informed consent. All participants completed an online screening form to determine whether they satisfied inclusion criteria. In particular, subjects were recruited if they were currently healthy (i.e. reported no current illness, psychiatric or otherwise), and reported no current drug use (including psychiatric drugs). Exclusion criteria were: a) treatment, diagnosis or medication for any psychiatric condition (lifetime), b) history of alcohol or substance dependence (lifetime) or recent abuse, or c) illegal drug use in the last month.

### Emotional working memory and decision-making task

On each trial of the task (Fig. 1B), participants were initially instructed to memorize the locations of two prime stimuli on the screen for 3 seconds, followed by a delay of 500 ms. Both prime stimuli belonged to one of the following four conditions: happy faces, fearful faces, neutral faces, or objects (light bulbs). A gamble then appeared for 2 s, during which participants had to decide whether to accept or reject the gamble, followed by another delay of 500 ms. Finally, one of the initial faces/objects from the pair appeared (memory probe), and participants were asked to indicate whether it had been located on the left or the right of the first screen. In total participants completed 392 trials, 49 in each of 8 conditions (4 prime conditions * 2 threat conditions). Block lengths ranged between 19 and 24 trials, so that participants were not able to anticipate when exactly a block would end.

### Stimuli

Face stimuli consisted of pictures from the NimStim Face Stimulus Set (http://www.macbrain.org/resources.htm). A set of 40 identities was chosen (20 male faces and 20 female faces), and each face stimulus was presented depicting either a neutral, happy, or fearful expression, resulting in a set of 120 face stimuli (primes). For the object control condition, 20 pictures of light bulbs were selected. All stimuli were resized to a resolution of 200 (width) × 300 (height) pixels and were displayed on a black background using Cogent 2000 (www.vislab.ucl.ac.uk/cogent.php) running under Matlab.

### State anxiety manipulation

Acute anxiety was induced by unpredictable electrical shocks delivered using a Digitimer DS7A Constant Current Stimulator (Digitimer Ltd., Welwyn Garden City, UK), with two electrodes secured to the wrist (as per Robinson *et al*.^10^). Each participant completed a shock thresholding procedure, consisting of a few (usually 2 to 5) electrical shocks of increasing intensity, to reach a level of ‘painful but tolerable’ i.e. 4 on a scale from 1 (no sensation) to 5 (painful and not tolerable).

Threat and safe blocks were indicated by an orange screen with ‘YOU ARE NOW AT RISK OF SHOCK’ or a blue screen with ‘YOU ARE NOW SAFE FROM SHOCK’ displayed for 3 seconds at the start of each block (Fig. 1B), and throughout the block by an orange or blue border, indicating threat or safe, respectively. A total of 6 shocks was delivered across the 9 threat blocks (see asterisks in Fig. 1A for approximate position of shock delivery throughout the task). On each of these six shock trials, the shock was either administered during the first or second fixation cross. Participants were informed that shocks would be unpredictable and independent of performance on the gambling and memory tasks.

### Procedure

Participants completed an online screening questionnaire as described above. If eligible, they were invited to the testing session. Participants first completed the Short State Anxiety Inventory (SSAI)^41^. They then practiced a 20-trial version of the emotional working memory task (without the concurrent gambling task) in which they had to memorise the position of the two faces presented on the screen, and then after a delay of 3 seconds, reported the previous location of the stimulus displayed on the centre of the screen. Participants then practiced 49 trials of the gambling task without the concurrent memory task. Participants were presented with mixed gambles with a 50% probability of winning an amount written in green, and 50% chance of losing an amount written in red, and had to decide whether to take (accept) the gamble or not (reject). These gambling-only trials were used to estimate each participant’s indifference point, such that the 7*7 gain/loss matrix for the main task could be centred on this indifference point in order to maximize sensitivity. Participants then practiced a 40-trial version of the task that included both the gambling and the emotional working memory components (equivalent to Fig. 1B but excluding the stress manipulation). The electrodes were then attached, the shock thresholding procedure was completed, and participants completed the 18 blocks of the main experiment (50 min). Payment was incentive-compatible, such that participants started the task with an initial £15, and the average outcome of 10 randomly selected choices (across the whole task) was added to the £15 to determine their final payment.

Participants provided self-report measures of depression (Beck Depression Inventory^54^; BDI) trait anxiety (STAI^11^).

### Statistical analysis

Matlab (www.mathworks.com) was used to extract and process the data, IBM SPSS Statistics (version 21) for frequentist analyses and JASP^55^,^56^ (version 0.7.1) for Bayesian analyses. Trials were excluded if the participant failed to respond to the gamble. The probability of accepting the gamble (P_accept_), mean reaction time (RT) to accept or reject the gamble, number of missed trials, and working memory accuracy and reaction times were calculated for each emotional condition (Happy, Fear, Neutral and Object) and threat condition (Threat, Safe) separately. Working memory scores were arcsine-transformed as they were skewed towards very high accuracy. Gamble acceptance rate and working memory performance were analysed using repeated-measures analyses of variance (ANOVAs) in order to assess the impact of (i) emotion prime condition, (ii) state anxiety manipulation (threat versus safe blocks) and (iii) their interaction. Trait anxiety scores were mean corrected and added as a continuous covariate (trait anxiety scores were normally distributed – Shapiro-Wilk test statistic: 0.980, *p* = 0.51). Significant interactions were followed up with post-hoc t-tests and/or Pearson’s correlations with trait anxiety.

Bayesian analyses were conducted to corroborate significant effects, as well as to provide evidence for null effects. Contrary to classical frequentist approaches, Bayesian analyses can provide evidence in favour of the null model, by showing that the data are better explained by the null model than any other models. The analysis compares different models of the data (e.g. including various combinations of main effects and/or interactions of interest) with the null model (model without main effect or interaction), allowing to draw inference about which model best explains the data. In practice, Bayesian ANOVAs, correlations, and t-tests were used to generate Bayes Factors (BF_10_), which represent the evidence for a model of interest relative to a null model (main effect of subject; 1 versus 0)^57^,^58^. A BF_10_ greater than 1 means that the model of interest performs better than the null model; a BF_10_ smaller than 1 constitutes evidence in favour of the null model. We used the magnitude of the BF_10_ as an index to interpret the strength of evidence^59^,^60^. Evidence in favour of the model of interest is considered anecdotal (1 < BF_10_ < 3), substantial (3 < BF_10_ < 10), strong (10 < BF_10_ < 30), very strong (30 < BF_10_ < 100) or decisive (BF_10_ > 100). Similarly, evidence in favour of the null model could also be qualified as anecdotal (0.33 < BF_10_ < 1), substantial (0.1 < BF_10_ < 0.33), strong (0.033 < BF_10_ < 0.1), very strong (0.01 < BF_10_ < 0.033) or decisive (BF_10_ < 0.01). When BF_10_ was greater than 100, we report its natural logarithm (ln(BF_10_)) for ease of interpretation. In order to compare the relative predictive success of one model over another, the BF_10_ of the first model was divided by the BF_10_ of the other model, and the value of this ratio interpreted in terms of strength of evidence using the same values as above.

### Loss aversion modelling

In order to assess loss aversion a model was estimated based on Prospect Theory’s subjective utility function^38^,^39^,^40^, using a maximum likelihood estimation procedure in Matlab. For each trial, the subjective utility (u) of each gamble was estimated using the following equation (with losses coded as negative values):


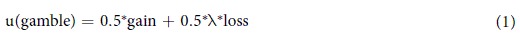


where λ is the “loss aversion” parameter: λ > 1 indicates an overweighing of gains relative to losses and λ < 1 the converse. These subjective utility values were used in a softmax function to estimate the probability of accepting each gamble (coded as 0 or 1 for each rejected or accepted gamble, respectively):


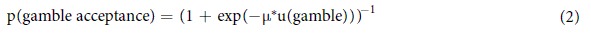


where μ is the logit sensitivity or “inverse temperature” parameter, an index of choice consistency for repeated identical gambles, equivalent to the maximal slope of a logistic regression curve: higher μ values indicate more consistent choices.

In order to assess whether loss aversion or choice consistency were altered by emotion or state anxiety, λ and μ values were estimated separately for each emotion condition and for safe and threat blocks and analysed similarly to gamble acceptance and working memory data. Due to high skewness values, μ values were square root transformed and λ values were log transformed before being entered into the ANOVAs.

## Additional Information

**How to cite this article**: Charpentier, C. J. *et al*. Anxiety promotes memory for mood-congruent faces but does not alter loss aversion. *Sci. Rep.* **6**, 24746; doi: 10.1038/srep24746 (2016).

## Figures and Tables

**Table 1:** Summary of effects of pathological anxiety disorders (A) and acute anxiety/stress induction (B) on risky decision-making.

A. *Patients with anxiety disorders*			
Study	Group	Task	Effect on risk takingvs healthy individuals
Maner *et al*.^23^ (study 3)	Anxiety disorders, Mood disorders, Learning/no Axis 1 disorders	RTBS (14-item version)	↓ in anxiety patients = in other groups
Mueller *et al*.^24^	GAD	IGT (modified)	↓ (specific to decisions with long-term loss)
Giorgetta *et al*.^27^	GAD, PAD	PGT (lotteries)	↓
Ernst *et al*.^28^	GAD, SocPh, SAD (all adolescents)	Loss aversion	=
Butler & Mathews^29^	GAD, MDD	Questionnaire	Overestimation of risk for negative events
**B.** ***Acute anxiety/stress induction in healthy individuals***			
**Study**	**Stressor**	**Task**	**Effect on risk taking**
Raghunathan & Pham^30^	Anxious mood induction	One-shot choice between 2 gambles	↓
Lighthall *et al*.^31^	CPT	BART	↑ in men ↓ in women
Mather *et al*.^32^	CPT	Driving task	↓ in older aduts = in younger adults
Porcelli & Delgado^33^	CPT	PGT (lotteries)	↓ in gain domain ↑ in loss domain
Putman *et al*.^34^	Administration of cortisol	PGT (lotteries)	↑ for high-risk gamble with large gain = otherwise
Clark *et al*.^26^	ToS (fear)	PGT (lotteries)	↓
Pabst *et al*.^35^	TSST	GDT (modified version)	= in gain domain ↓ in loss domain
Buckert *et al*.^36^	TSST	PGT (lotteries)	↑ in gain domain ↓ in loss domain
Robinson *et al*.^10^	ToS (anxiety)	IGT	↓ in low trait anxious ↑ in high trait anxious
Robinson *et al*.^37^	ToS (anxiety)	Framing effect Temporal discounting	=

↑ means increased risk taking; ↓ decreased risk taking, = no effect. CPT: Cold Pressor Test, ToS: Threat of Shock, TSST: Trier Social Stressor Test, BART: Balloon Analogue Risk Task, PGT: Probabilistic Gambling Task, GDT: Game of Dice Task, IGT: Iowa Gambling Task, RTBS: Risk-Taking Behaviors Scale, GAD: Generalized Anxiety Disorder, PAD: Panic Attack Disorder, MDD: Major Depressive Disorder, SocPh: Social Phobia, SAD: Separation Anxiety Disorder.

**Table 2:** Demographic data and questionnaire scores.

N = 55	Mean (SD)
*Age (years)*	24.15 (5.59)
*Gender ratio (m:f)*	24:31
*BDI-II (total)*	6.00 (6.98)
*STAI (Trait)*	38.92 (8.76)
*SSAI prior to testing*	10.16 (3.35)
*SSAI for safe blocks*	10.92 (3.37)
*SSAI for threat blocks*	15.98 (3.39)

BDI-II: Beck Depression Inventory II; STAI: State Trait Anxiety Inventory; SSAI: Short State Anxiety Inventory. SSAI was administered before the task, as well as after the task with questions phrased retrospectively about the safe blocks and the threat blocks.

**Table 3:** Summary of cognitive outcome variables (N = 55).

Mean(SD)	Safe Happy	Safe Fearful	Safe Neutral	Safe Object	Threat Happy	Threat Fearful	Threat Neutral	Threat Object
Working memory (proportion correct)	0.9271 (0.086)	0.9226 (0.094)	0.9295 (0.096)	0.8986 (0.102)	0.9313 (0.089)	0.9219 (0.089)	0.9237 (0.096)	0.9190 (0.090)
Gambling (proportion accepted)	0.4713 (0.118)	0.4616 (0.125)	0.4684 (0.125)	0.4834 (0.127)	0.4760 (0.120)	0.4664 (0.123)	0.4632 (0.121)	0.4820 (0.125)
Loss aversion (λ parameter)	1.9308 (1.329)	1.9427 (1.287)	1.9341 (1.375)	1.8876 (1.231)	1.9137 (1.362)	1.9123 (1.257)	1.9352 (1.319)	1.9081 (1.396)
Choice consistency (μ parameter)	2.8746 (1.958)	2.5142 (1.882)	2.6830 (1.867)	2.6455 (1.809)	2.7328 (1.915)	2.4924 (1.816)	2.8493 (1.950)	2.5894 (1.855)
Working memory reaction times (in seconds)	0.7453 (0.013)	0.7481 (0.017)	0.7428 (0.016)	0.7796 (0.015)	0.7305 (0.014)	0.7332 (0.014)	0.7346 (0.014)	0.7635 (0.015)
Gambling reaction times (in seconds)	0.9519 (0.172)	0.9538 (0.168)	0.9480 (0.168)	0.9581 (0.156)	0.9387 (0.164)	0.9407 (0.159)	0.9406 (0.164)	0.9434 (0.166)

Means (standard deviations) are reported for each experimental condition and each dependent variable (untransformed) analysed in the study.
